# Projections of anxiety disorder prevalence during and beyond the COVID-19 pandemic in Germany using the illness–death model

**DOI:** 10.1192/bjo.2024.754

**Published:** 2024-10-10

**Authors:** Chisato Ito, Bernhard T. Baune, Tobias Kurth, Ralph Brinks

**Affiliations:** Institute of Public Health, Charité – Universitätsmedizin Berlin, Berlin, Germany; Department of Psychiatry, University of Münster, Münster, Germany; Department of Psychiatry, Melbourne Medical School, University of Melbourne, Melbourne, Victoria, Australia; and Florey Institute of Neuroscience and Mental Health, University of Melbourne, Melbourne, Victoria, Australia; Faculty of Health/School of Medicine, Witten/Herdecke University, Witten, Germany; and Institute for Biometrics and Epidemiology, German Diabetes Center, Leibniz Center for Diabetes Research at Heinrich Heine University, Düsseldorf, Germany

**Keywords:** Anxiety disorders, COVID-19, prevalence, incidence, epidemiology

## Abstract

**Background:**

Although there is now substantial evidence on the acute impacts of the COVID-19 pandemic on anxiety disorders, the long-term population impact of the pandemic remains largely unexplored.

**Aims:**

To quantify a possible longitudinal population-level impact of the pandemic by projecting the prevalence of anxiety disorders through 2030 among men and women aged up to 95 years in Germany under scenarios with varying impacts of the pandemic on the incidence of anxiety disorders.

**Method:**

We used a three-state illness–death model and data from the Global Burden of Disease Study to model historical trends of the prevalence and incidence of anxiety disorders. The German population projections determined the initial values for projections. The COVID-19 incidence rate data informed an additional incidence model, which was parameterised with a wash-in period, delay, wash-out period, incidence increase level and decay constant.

**Results:**

When no additional increase in the incidence during the pandemic waves during 2020–2022 was assumed, it was estimated that 3.86 million women (9.96%) and 2.13 million men (5.40%) would have anxiety disorders in 2030. When increases in incidence following pandemic waves were assumed, the most extreme scenario projected 5.67 million (14.02%) women and 3.30 million (8.14%) men with the mental disorder in 2030.

**Conclusions:**

Any increased incidence during the pandemic resulted in elevated prevalence over the projection period. Projection of anxiety disorder prevalence based on the illness–death model enables simulations with varying assumptions and provides insight for public health planning. These findings should be refined as trend data accumulate and become available.

Anxiety disorders impose a large burden on the global population,^1^ with an estimated prevalence of 301 million globally (4.1%) and 5.7 million (7.1%) in Germany in 2019.^2^ Anxiety disorders are characterised by excessive, persistent and function-impairing fear and anxiety of perceived threats, with subtypes such as specific phobias, panic disorder, social anxiety disorder and generalised anxiety disorder; they disproportionately affect women and are often undertreated.^3–5^

## COVID-19 and anxiety disorders

The emergence of the coronavirus disease (COVID-19) pandemic in 2020 highlighted the potential mental health impacts of both the virus itself and pandemic control measures, and these were quickly identified as urgent public health concerns.^6^ COVID-19 can directly (i.e. as neuropsychiatric sequelae of COVID-19) and indirectly (i.e. via societal changes due to pandemic control measures) negatively affect mental health and has increased anxiety and depression symptoms, although findings vary greatly, with many methodological limitations.^7^ For instance, the Global Burden of Disease (GBD) Study reported a 25.6% increase in the prevalence of anxiety disorders globally during 2020, based on 27 eligible studies identified through a systematic review.^8^ Conversely, a living systematic review of studies comparing time periods before and during the pandemic reported minimal changes in mental health symptoms.^9^

Although inconclusive, the growing evidence has shed light on the immediate effects of the COVID-19 pandemic on anxiety disorders, which may be magnified if new cases of anxiety disorders are not identified and intervened in appropriately. The long-term mental health consequences of the pandemic at the population level, however, remain uncertain and require investigation. The need for a better understanding of the longitudinal population-level mental health impact of the pandemic is underscored by the increasing number of infectious disease outbreaks globally.^10^ Determining how long the mental health effects of COVID-19 may last and quantifying such impacts in terms of the prevalence of anxiety disorders could provide insights for future emergency plans and resource allocation for psychological and psychiatric care.

## Illness–death model

Multistate modelling provides a structure for projecting long-term disease burden in a specific country or region where general population fluctuations can be estimated. Although multistate models are commonly used in infectious disease epidemiology, their use in chronic disease research is still emerging.^11,12^ The illness–death model, which compartmentalises a population into different health states and relates incidence, prevalence, remission and mortality, can be applied to a wide range of chronic diseases, including psychiatric disorders.^13^

Our study aimed to estimate the burden of the COVID-19 pandemic on anxiety disorders in terms of disease prevalence during and beyond the pandemic, by projecting the numbers of men and women aged up to 95 years with anxiety disorders in Germany from 2019 to 2030, using the illness–death model and simulating varying patterns of anxiety disorder incidence following the pandemic waves.

## Method

### Data sources

#### Epidemiological data of anxiety disorders

We obtained age- and sex-specific incidence and prevalence estimates for anxiety disorders in Germany from 1990 to 2019 from the GBD study.^14^ These estimates are a combined estimate of all anxiety disorder subtypes, with ICD codes F40–42, F43.0, F43.1, F93.0–93.2 and F93.8 and DSM-IV-TR codes 300.0–300.3, 308.3, 309.21 and 309.81.^1^

#### Population projection

The German population projections by age and sex up to 2070 were obtained from the Federal Statistical Office of Germany.^15^ From a total of 27 variations of the population projections provided, we used the variant 2 projection, which assumed a stable fertility rate, moderate increase in life expectancy and moderate net migration.^15^ From these population growth estimates, we drew the initial values and expected fluctuations due to both birth and death for our projection.

#### COVID-19 incidence

Weekly COVID-19 incidence rates from the onset of the pandemic in 2020 to the end of 2022 were obtained from SurvStat@RKI 2.0, a database maintained by the Robert Koch Institute (RKI), the governmental public health institute of Germany.^16^

### Statistical analysis

**Fig. 1:**
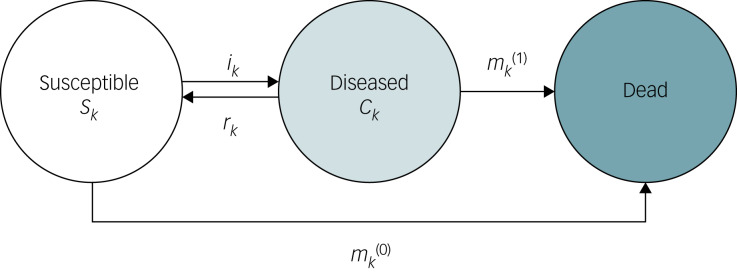
Illness–death model. The population under consideration is divided into three compartments: susceptible *S_k_*, diseased *C_k_* and dead. Arrows between the states indicate possible transitions: incidence rate *i_k_*, mortality rate among the susceptible 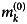, mortality rate among the diseased 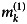 and remission probability among the diseased *r_k_*.

We applied a three-state illness–death model with discrete time steps, in which individuals within a population transitioned between the ‘susceptible,’ ‘diseased’ and ‘dead’ states (Fig. 1). In irreversible chronic diseases, the relevant rates of transition from one state to another are the incidence *i*, the mortality rate among the susceptible *m*^(0)^ and the mortality rate among the diseased *m*^(1)^. However, although anxiety disorders can be long-lasting, remission is often possible and is a desired treatment outcome.^3,17^ Consequently, the inclusion of the remission probability, *r*, where the diseased move back to the susceptible state, is crucial for understanding the epidemiology of anxiety disorders.

#### Notation

We denote populations in the susceptible and diseased compartments of the illness–death model as follows:

- *S_k_* = number of individuals at time *t_k_* in the susceptible state;
- *C_k_* = number of individuals at time *t_k_* in the diseased state.

Further, we denote transition rates between the three states as follows:

- *i_k_* = incidence rate;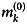 = mortality rate among susceptible individuals;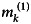 = mortality rate among individuals with the disease;
- *r_k_* = remission probability among individuals with the disease;

where *t_k_* = *k_τ_* with *τ* > 0, *k =* 0, 1, …

*S*_*k*_ and *C*_*k*_ at time *t*_*k* + 1_ and at the preceding time *t*_*k*_ can be expressed using difference equations as1
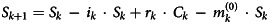
2
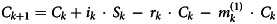


The disease prevalence *p* at time *t*_*k* + 1_ can in turn be expressed as
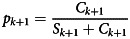
and the prevalence odds *π* at time *t_k + 1_* is3
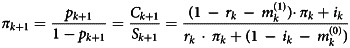


The derived recursion formula (Eq. 3) allows us to estimate the prevalence odds *π* at time *t_k + 1_* based on the preceding time point *t_k_*. Repeated application of this formula allows us to make a projection of the prevalence odds, or another transition rate of interest, into the future for a specified period. Further details of the derivation are provided elsewhere.^13^ The age-stratified versions of these formulae were used for our analyses.

#### Estimation of transition rates

We fitted natural cubic spline models to the historical incidence and prevalence estimates from the GBD study as a function of age and time for each sex group. Currently, no age- or sex-specific mortality rates for anxiety disorders in Germany are available. However, a meta-analysis indicated a 1.4-fold all-cause mortality risk among individuals with anxiety disorders compared with those without.^18^ Hence, we modelled the mortality rates among the diseased and the susceptible linearly, reflecting the mortality risk ratio of 1.4 and incorporating our assumption of a gradual decrease in mortality risk over time with an expected improvement in the disease and comorbidity management. Further, the rate of decrease in the mortality risk over time can be expected to be more rapid in the disease group, as has been observed in other chronic diseases.^19^ In terms of remission, to our knowledge, there is no existing evidence on the age- or sex-specific probability of remission for patients with anxiety disorders. Given the prevalence, incidence and mortality estimates, however, we estimated the remission probability by the recursive application of Eq. 3 solved for *r_k_*:4
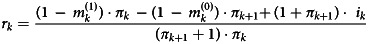


By combining the estimated remission probability with the incidence and mortality rates, we estimated the number of individuals in each of the illness–death model compartments at a specific time point and into the future, given a starting value for each state. Such estimates constitute a projection of anxiety disorder cases and thus the prevalence of anxiety disorders in the German population when respective transition rates (i.e. incidence, mortality and remission) follow historical trends. Essentially, these projections show what the prevalence of anxiety disorders might have been if the COVID-19 pandemic had had no impact on them (scenario 0).

#### Modelling increased anxiety incidence following pandemic waves

An increase in anxiety and depressive symptoms has been observed to follow the increase in COVID-19 cases, with a noticeable spike in symptoms in the early months of the pandemic.^20,21^ We therefore modelled an increase in the incidence of anxiety disorders that followed the pandemic waves, which was then added to the baseline incidence model to project disease cases from the onset of the pandemic in 2020 to 2030. Hereafter, we describe how we parameterised this additional incidence model.

By the end of 2022, six pandemic waves were reported to have passed,^22^ and the RKI COVID-19 incidence rate data demonstrated an additional seventh wave at the end of 2022. Wave peaks were identified by fitting multiple Gaussians to the COVID-19 incidence rate data, and wave durations were approximated by the full width at half maximum (FWHM) of each wave peak. The FWHM for a Gaussian function can be calculated by 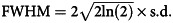 where s.d. is the estimated standard deviation.^23^

We then modelled the anxiety disorder incidence to rise linearly during each wave, using the following modelling parameters with deterministic assumptions: wash-in and wash-out period, delay, incidence increase level and decay (Supplementary Figure 1). The wash-in period *W_in_* characterises the period from the start of a given pandemic wave to the time when the anxiety disorder incidence peaked during the wave, and the wash-out period *W*_out_ represents the time between the point the elevated anxiety disorder incidence started to decline and the point when the incidence was no longer elevated above what would be expected from historical trends before the pandemic (*W*_in_ = *W*_out_ = 0.1, 0.2 or 0.3). For each wave, a delay *Δ* was introduced, during which the COVID-19 incidence started to subside, whereas the anxiety disorder incidence remained elevated (*Δ* = 0.0, 0.25 or 0.5). The magnitude of the increase in anxiety disorder incidence at the peak, *h*_0_ = 1.0, 5.0 or 10.0, was set to gradually diminish from one wave to another, at a rate of exponential decay *λ* = 0.1, 0.2 or 0.3, assuming individual adaptation to societal changes and uncertainty, less stringent restriction measures, and increased prevention and treatment options. We then ran projections for combinations of these model parameters (*W*_in_, *W*_out_, *Δ*, *h*_0_ and *λ*) from 2019 to 2030. All 82 simulated scenarios and combinations of the incidence model parameters are summarised in Supplementary Table 1 available at https://doi.org/10.1192/bjo.2024.754. The projection period was chosen to cover the critical initial years of the pandemic and the following years of transition to long-term management.

### Software

All analyses were performed using R 4.2.1 and RStudio 2023.06.0+421.^24,25^ The analysis code is publicly available at https://github.com/chisato-ito/idm_anxiety_disorders.

### Ethics approval

This study is a secondary analysis of publicly available and anonymised data-sets containing no individually identifiable data and thus required no additional ethics approval.

## Results

**Fig. 2:**
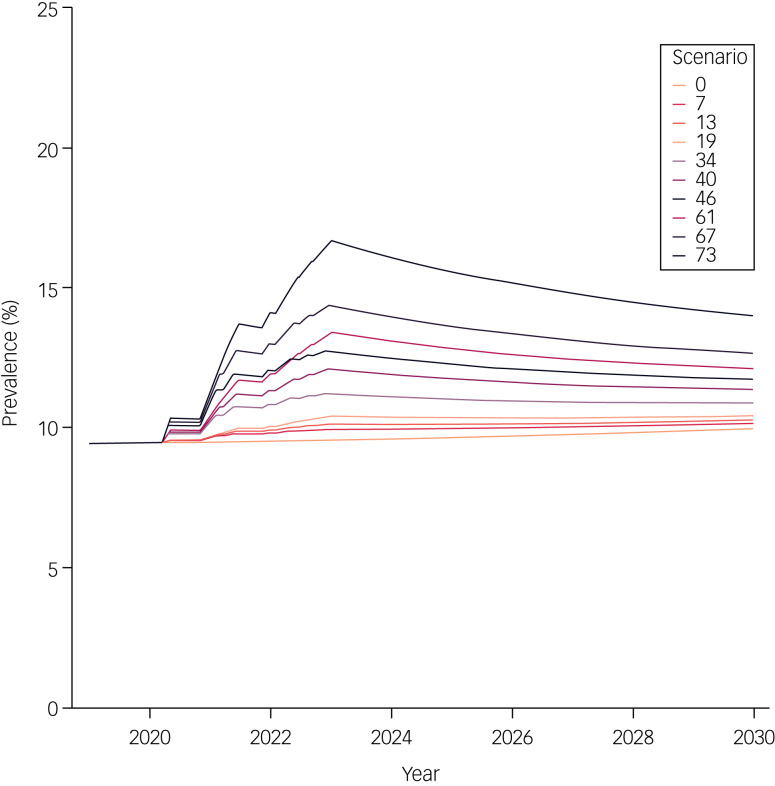
Projected prevalence of anxiety disorders among women in Germany from 2019 to 2030. Prevalence (%) of anxiety disorders among women in Germany from 2019 to 2030, projected with the illness–death model under ten selected scenarios. In scenarios 7, 13 and 19, the extent of the increase in incidence at its peak *h*_0_ was set to 1. Scenario 7 assumed a large delay and a small decay constant (*Δ* = 0.5; *λ* = 0.1); scenario 13 mimicked a moderate delay and decay (*Δ* = 0.25; *λ* = 0.2) and scenario 19 simulated no delay and a large decay constant (*Δ* = 0; *λ* = 0.3). In scenarios 34, 40 and 46, *h*_0_ was set to 5. We provided scenario 34 with a large delay and a small decay constant (*Δ* = 0.5; *λ* = 0.1), scenario 13 with a moderate delay and decay (*Δ* = 0.25; *λ* = 0.2) and scenario 19 with no delay and a large decay (*Δ* = 0; *λ* = 0.3). In scenarios 61, 67 and 73, *h*_0_ was set to 10. We provided scenario 61 with a large delay and a small decay constant (*Δ* = 0.5; *λ* = 0.1), scenario 67 with a moderate delay and decay (*Δ* = 0.25; *λ* = 0.2), and scenario 73 with no delay and a large decay (*Δ* = 0; *λ* = 0.3).

**Fig. 3:**
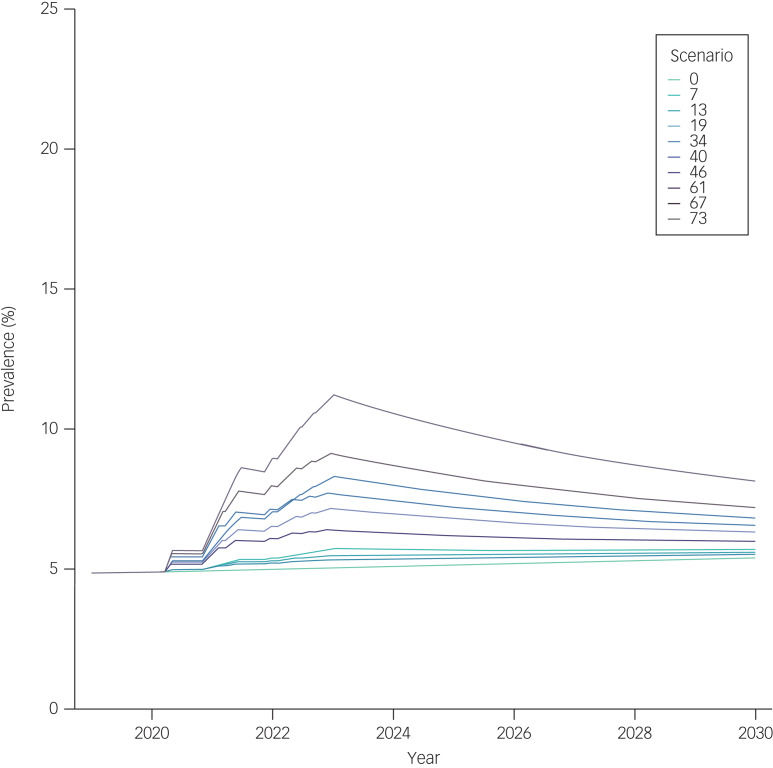
Projected prevalence of anxiety disorders among men in Germany from 2019 to 2030. Prevalence (%) of anxiety disorders among men in Germany from 2019 to 2030, projected with the illness–death model under ten selected scenarios. In scenarios 7, 13 and 19, the extent of the increase in incidence at its peak *h*_0_ was set to 1. Scenario 7 assumed a large delay and a small decay constant (*Δ* = 0.5; *λ* = 0.1); scenario 13 mimicked a moderate delay and decay (*Δ* = 0.25; *λ* = 0.2) and scenario 19 simulated no delay and a large decay constant (*Δ* = 0; *λ* = 0.3). In scenarios 34, 40 and 46, *h*_0_ was set to 5. We provided scenario 34 with a large delay and a small decay constant (*Δ* = 0.5; *λ* = 0.1), scenario 13 with a moderate delay and decay (*Δ* = 0.25; *λ* = 0.2) and scenario 19 with no delay and a large decay (*Δ* = 0; *λ* = 0.3). In scenarios 61, 67 and 73, *h*_0_ was set to 10. We provided scenario 61 with a large delay and a small decay constant (*Δ* = 0.5; *λ* = 0.1), scenario 67 with a moderate delay and decay (*Δ* = 0.25; *λ* = 0.2) and scenario 73 with no delay and a large decay (*Δ* = 0; *λ* = 0.3).

Figures 2 and 3 show the projected prevalence of anxiety disorders in Germany from 2019 to 2030 among women and men, respectively. Supplementary Figures 2 and 3 show the projected numbers of anxiety disorder cases among women and men, respectively. The results of ten selected scenarios, of a total of 82, are presented in Table 1. Variations in the *W*_in_ and *W*_out_ parameters resulted in relatively small changes in the results compared with those that occurred when *Δ*, *h*_0_ and *λ* were varied. Therefore, in scenarios 7, 13, 19, 34, 40, 46, 61, 67 and 73, which are shown in Figs. 2 and 3 and Supplementary Figs. 2 and 3, *W*_in_ and *W*_out_ were kept constant at 0.10; that is, both the wash-in period and wash-out period were assumed to be 10% of each pandemic wave duration.

**Table 1:** Projected numbers of cases and prevalence of anxiety disorders in 2020, 2021, 2023 and 2030 in Germany, by sex and scenario

Estimated numbers of cases and prevalence of anxiety disorders in Germany by sex													
						2020		2021		2023		2030	
Scenario	*W*_in_	*W*_out_	*Δ*	*λ*	*h*_0_	Cases (millions)	Prevalence (%)	Cases (millions)	Prevalence (%)	Cases (millions)	Prevalence (%)	Cases (millions)	Prevalence (%)
Women													
0	0.00	0.00	0.00	0.00	0.00	3.900	9.47	3.887	9.49	3.867	9.56	3.860	9.96
7	0.10	0.10	0.50	0.10	1.00	3.900	9.47	3.969	9.69	4.226	10.42	4.051	10.44
13	0.10	0.10	0.25	0.20	1.00	3.900	9.47	3.959	9.67	4.098	10.14	3.983	10.28
19	0.10	0.10	0.00	0.30	1.00	3.900	9.47	3.950	9.65	4.014	9.95	3.938	10.18
34	0.10	0.10	0.50	0.10	5.00	3.900	9.47	4.297	10.41	5.614	13.39	4.790	12.11
40	0.10	0.10	0.25	0.20	5.00	3.900	9.47	4.248	10.30	5.002	12.10	4.462	11.38
46	0.10	0.10	0.00	0.30	5.00	3.900	9.47	4.201	10.20	4.591	11.22	4.246	10.89
61	0.10	0.10	0.50	0.10	10.00	3.900	9.47	4.703	11.28	7.254	16.64	5.665	14.02
67	0.10	0.10	0.25	0.20	10.00	3.900	9.47	4.605	11.07	6.090	14.36	5.041	12.66
73	0.10	0.10	0.00	0.30	10.00	3.900	9.47	4.512	10.87	5.296	12.72	4.622	11.74
Men													
0	0.00	0.00	0.00	0.00	0.00	1.974	4.89	1.988	4.93%	2.017	5.02	2.128	5.40
7	0.10	0.10	0.50	0.10	1.00	1.974	4.89	2.054	5.09%	2.305	5.72	2.250	5.70
13	0.10	0.10	0.25	0.20	1.00	1.974	4.89	2.046	5.08%	2.201	5.48	2.206	5.59
19	0.10	0.10	0.00	0.30	1.00	1.974	4.89	2.039	5.06%	2.133	5.32	2.177	5.52
34	0.10	0.10	0.50	0.10	5.00	1.974	4.89	2.320	5.72%	3.430	8.28	2.726	6.82
40	0.10	0.10	0.25	0.20	5.00	1.974	4.89	2.280	5.62%	2.927	7.16	2.511	6.32
46	0.10	0.10	0.00	0.30	5.00	1.974	4.89	2.242	5.53%	2.594	6.39	2.371	5.99
61	0.10	0.10	0.50	0.10	10.00	1.974	4.89	2.650	6.48%	4.780	11.18	3.297	8.14
67	0.10	0.10	0.25	0.20	10.00	1.974	4.89	2.571	6.29%	3.811	9.12	2.882	7.19
73	0.10	0.10	0.00	0.30	10.00	1.974	4.89	2.495	6.12%	3.160	7.68	2.609	6.55

*W*_in_, wash-in period, a period from the start of a given pandemic wave to the time when the anxiety disorder incidence peaks during the wave; *W*_out_, wash-out period, a time between the point when the elevated anxiety disorder incidence starts to decline and that when the incidence is no longer elevated above expected based on historical trends; *Δ*, delay, during which the COVID-19 incidence starts to subside, while the anxiety disorder incidence remains elevated; *h*_0_, the magnitude of the increase in the incidence of anxiety disorders at the peak; *λ*, decay constant at which *h*_0_ gradually diminishes from one wave to another.

At the start of 2020, before COVID-19 was declared a pandemic, the estimated number of cases of anxiety disorders were 3.90 million (prevalence: 9.47%) among women and 1.97 million (prevalence: 4.89%) among men (Table 1).

In our baseline projection (scenario 0), we assumed that all transition rates in the illness–death model would follow historical trends even after the start of the pandemic, with no sudden spikes in incidence occurring during the pandemic waves. Under this scenario, 3.87 million (9.56%) women and 2.02 million (5.02%) men were estimated to have anxiety disorders in 2023. Then, at the end of the projection period in 2030, we estimated 3.86 million cases (9.96%) in women, a slight decrease in the absolute number but a slight increase in the prevalence; and 2.13 million cases (5.40%) in men, an increase in both the absolute number and the prevalence over time.

We observed the smallest change in anxiety disorder cases and prevalence compared with baseline in scenario 19, where *Δ* was set to 0 (i.e. the start of decline in anxiety disorder incidence aligned with the end of the pandemic wave as defined by FWHM), *λ* = 0.3 and *h*_0_ was set to 1 (i.e. a maximum 100% increase in incidence). In this scenario, after the seven pandemic waves in 2023, 4.01 million women (9.95%) and 2.13 million men (5.32%) would have anxiety disorders, compared with 3.94 million women (10.18%) and 2.18 million men (5.52%) in 2030.

The most extreme changes were observed in scenario 61, where *Δ* = 0.5, *λ* = 0.1 and *h*_0_ = 10. Under scenario 61, in 2023, 7.25 million (16.64%) women and 4.78 million (11.18%) men were estimated to suffer from anxiety disorders. The estimated values for 2030 were much lower than the peak values for 2023, with 5.67 million (14.02%) women and 3.30 million (8.14%) men expected to have anxiety disorders in this scenario.

Under scenarios 1–81, in both sexes, the estimated numbers of cases and prevalence remained elevated compared with the baseline projection (scenario 0) through 2030. Results for all 82 scenarios are provided in Supplementary Table 2.

## Discussion

We aimed to estimate the impact of the COVID-19 pandemic on mental health by analysing the sex-specific prevalence of anxiety disorders in Germany, from 2019 (pre-pandemic) through the onset of the pandemic in 2020, to 2030. We used a three-state illness–death model with discrete time steps to project numbers of individuals with anxiety disorders over this period. To account for the varying impact of the pandemic, we considered multiple scenarios with different modelling parameters to simulate different patterns of increase in anxiety incidence following the seven pandemic waves from 2020 to 2022.

In our baseline projection, we assumed that the age- and sex-specific incidence of anxiety disorders would simply follow the historical trend, unaffected by the pandemic. The results showed a slight decrease in the absolute number of disease cases in women and a slight increase in men, as well as a small (<1%) increase in the prevalence in both sexes, by the end of the projection period of 2030 compared with the start of 2020. However, in the 81 alternative scenarios in which we modelled temporary incidence increases of different magnitudes, durations and decay around the pandemic waves, the increases in numbers of cases and prevalence persisted throughout the projection period (Supplementary Table 2).

We set one of the modelling parameters, the extent of increase in anxiety disorder incidence at its peak, *h*_0_, to 1, 5 and 10. These values equate to +100%, +500% and +1000% increases in incidence, respectively. Although these values may appear quite extreme, it was only when *h*_0_ = 10 (scenario 61 in Figs. 2 and 3 and Supplementary Figs. 2 and 3) that we observed an approximately 25% increase in the prevalence of anxiety disorders for the total population at the beginning of 2021. This increase aligns with the rise attributed to the COVID-19 pandemic by the GBD study in 2021,^8^ which estimated a 25.6% (95% uncertainty interval 23.2–28.0) change in global prevalence of anxiety disorders due to the pandemic in a meta-regression based on data from 27 studies published between January 2020 and January 2021.^8^ At the end of 2020, Germany was only in the midst of the second pandemic wave; this was followed by five more waves in the subsequent 2 years.^22^ If the incidence of anxiety disorders continued to rise during these following waves, as projected in scenario 61, we would anticipate a far greater disease burden by 2030, with approximately 5.67 million women (prevalence: 14.02%) and 3.30 million men (prevalence: 8.14%) affected by anxiety disorders. However, it is important to highlight that the currently available evidence on the relationship between the COVID-19 pandemic and mental health is mixed and sometimes contradictory.^7,9^ For instance, Sun et al^9^ reported no negative changes in general mental health (standardised mean difference (SMD) 0.11, 95% CI −0.00 to 0.22) or anxiety symptoms (SMD 0.05, 95% CI −0.04 to 0.13) between pre-COVID-19 and pandemic periods in their meta-analyses, which included 11 cohorts for general mental health and four cohorts for anxiety symptoms. As such, a large increase in the prevalence of anxiety disorders, as projected in scenario 61, may be too extreme and possibly unrealistic. By contrast, our projection results in which the magnitude of the incidence increase at its peak was set *h*_0_ = 5 and *h*_0_ = 1 provide more moderate to conservative estimates of the anxiety disorder burden trajectory in Germany. Furthermore, we assumed that the remission probability would remain stable, which may have resulted in conservative estimates, as incident cases of anxiety disorders during pandemic waves could have had a lower probability of remission if they were not identified and treated in an appropriate and timely manner. In addition, it is important to consider the projected increase in the prevalence of anxiety disorders in conjunction with other changes due to the pandemic, such as care utilisation and resource availability, as the current evidence points to stable emergency department visits but an increase in use of telehealth care.^26,27^

For this study, we modelled the additional incidence of anxiety disorders to follow the COVID-19 incidence waves. This was based on empirical evidence showing increases and decreases in anxiety symptoms following the fluctuations of SARS-CoV-2 infections.^21^ We took a deterministic approach to model the rise and decline of incidence linearly. We focused on a small set of parameters, namely the wash-in period, delay, wash-out period, increase in incidence and decay constant, given our exploratory aim and limited understanding of any direct influence of COVID-19 on anxiety disorders. Further, we designed the models in such a way that the magnitude of increase in the anxiety disorder incidence would diminish gradually with variation in the decay constant *λ*. This assumed that individuals adapted to the pandemic and related societal changes, given the evidence of resilience seen in various populations.^28^ As more population-level data on anxiety disorders during the pandemic become available, and as we gain a clearer understanding of the link between COVID-19 and anxiety disorders, future work should focus on testing and refinement of the model.

Our study had several limitations. First, the sex- and age-specific prevalence and incidence rate estimates from the GBD study we used are not primary data but estimates based on thousands of data sources evaluated using a Bayesian meta-regression tool DisMod-MR.^1^ Although there may be concerns about the use of this method in data-scarce regions, it is less problematic for well-documented areas such as Germany. Second, the meta-analysis we used for the mortality rates^18^ was not tailored to specific demographics such as sex and age, or country. Given the sex disparity in anxiety disorders, more detailed data would improve the model's performance. The limited availability of mental health data in Germany also poses a challenge. A national effort by the RKI to establish a mental health surveillance system is underway.^29^ Third, we assumed that mortality rates would decrease more rapidly among those with anxiety disorders than among the susceptible over time, based on trends observed in other non-psychiatric diseases such as diabetes.^19^ Although we must be cautious in making assumptions about the disease course of a mental disorder based on non-psychiatric conditions, a decreasing trend in mortality rates can be reasonably assumed for those with anxiety disorders if we expect the treatment and the management of the disorders, as well as other important aspects such as somatic comorbidity and substance use, to improve over time. Continued efforts to build on our current understanding of anxiety disorders and mortality rates^18,30^ are required to better inform the magnitude of such a decline. Fourth, our modelling of anxiety disorder incidence involved several assumptions. For instance, although we linked anxiety symptoms to SARS-CoV-2 infection waves, an increase in mental health symptom levels do not necessarily equate to an increase in clinical cases. Moreover, the mental health impact of the pandemic varies based on factors including age, sex and education.^7^ People with pre-existing mental illness may also be disproportionately affected by the pandemic.^31^ The proposed illness–death model does not account for this diversity, treating everyone in the same state equally. However, the scenarios we simulated provide a range of macro-level transition rate estimates, which can be tested against when more data become available. Fifth, we assumed that the prevalence of anxiety disorders was comparable between the resident and migrant (both immigrant and emigrant) populations. It has been shown that changes in disease prevalence over time do not depend on migration when the prevalence among the two groups is the same; moreover, even if the prevalence differs between the two populations differs, if the number of migrants is small, the impact of migration on prevalence will be negligible.^11^ However, given existing inequalities in mental healthcare access between migrants and non-migrants and the recent influx of refugees (who generally bear a greater mental health burden) due to the Russian invasion of Ukraine, further research on the mental health of migrant populations is necessary.^32,33^

Our approach to using the discrete time-step illness–death model allows forecast of the prevalence of anxiety disorders under various scenarios. We demonstrated its feasibility to estimate transition rates and characterise pandemic waves with publicly available data and relative ease of application. This modelling approach could be extended to other mental disorders. The results revealed that even a small, temporary increase in the incidence of anxiety disorders during pandemic waves could lead to a sustained increase in disease burden for many years beyond the pandemic itself. Although interpretation of the results requires caution owing to our limited understanding of the actual trends in anxiety disorder epidemiology following the COVID-19 outbreak in Germany, the projections could serve as a vital tool for mental healthcare resource planning and policy formulation. Continued efforts to gather mental health data and iterative refinement of the model are essential for a more accurate understanding of the distribution of anxiety disorders and the longitudinal population impacts of the COVID-19 pandemic.

## Supporting information

Ito et al. supplementary material Ito et al. supplementary material

## Data Availability

The data-sets analysed for this study are publicly available and can be found at the GBD Results Tool (https://vizhub.healthdata.org/gbd-results/), SurvStat@RKI 2.0 of the RKI (https://survstat.rki.de/) and the Federal Statistical Office (Destatis; https://service.destatis.de/bevoelkerungspyramide/). The analysis code is publicly available (https://github.com/chisato-ito/idm_anxiety_disorders).

## References

[1] GBD 2019 Mental Disorders Collaborators. Global, regional, and national burden of 12 mental disorders in 204 countries and territories, 1990–2019: a systematic analysis for the Global Burden of Disease Study 2019. Lancet Psychiatry 2022; 9: 137–50. 35026139 10.1016/S2215-0366(21)00395-3 PMC8776563

[2] Institute for Health Metrics and Evaluation. *GBD Results*. 2020 (https://vizhub.healthdata.org/gbd-results/ [cited 21 Mar 2023]).

[3] Penninx BWJH, Pine DS, Holmes EA, Reif A. Anxiety disorders. Lancet 2021; 397: 914–27. 33581801 10.1016/S0140-6736(21)00359-7 PMC9248771

[4] Bandelow B, Michaelis S. Epidemiology of anxiety disorders in the 21st century. Dialogues Clin Neurosci 2015; 17: 327–35. 26487813 10.31887/DCNS.2015.17.3/bbandelow PMC4610617

[5] Alonso J, Liu Z, Evans-Lacko S, Sadikova E, Sampson N, Chatterji S, et al. Treatment gap for anxiety disorders is global: results of the World Mental Health Surveys in 21 countries. Depress Anxiety 2018; 35: 195–208. 29356216 10.1002/da.22711 PMC6008788

[6] Holmes EA, O'Connor RC, Perry VH, Tracey I, Wessely S, Arseneault L, et al. Multidisciplinary research priorities for the COVID-19 pandemic: a call for action for mental health science. Lancet Psychiatry 2020; 7: 547–60. 32304649 10.1016/S2215-0366(20)30168-1 PMC7159850

[7] Penninx BWJH, Benros ME, Klein RS, Vinkers CH. How COVID-19 shaped mental health: from infection to pandemic effects. Nat Med 2022; 28: 2027–37. 36192553 10.1038/s41591-022-02028-2 PMC9711928

[8] COVID-19 Mental Disorders Collaborators. Global prevalence and burden of depressive and anxiety disorders in 204 countries and territories in 2020 due to the COVID-19 pandemic. Lancet 2021; 398: 1700–12. 34634250 10.1016/S0140-6736(21)02143-7 PMC8500697

[9] Sun Y, Wu Y, Fan S, Dal Santo T, Li L, Jiang X, et al. Comparison of mental health symptoms before and during the COVID-19 pandemic: evidence from a systematic review and meta-analysis of 134 cohorts. BMJ 2023; 380: e074224. 36889797 10.1136/bmj-2022-074224 PMC9992728

[10] Global Preparedness Monitoring Board. A World at Risk: Annual Report on Global Preparedness for Health Emergencies. Global Preparedness Monitoring Board, 2019 (https://apps.who.int/gpmb/assets/annual_report/GPMB_Annual_Report_English.pdf).

[11] Brinks R, Landwehr S. Age- and time-dependent model of the prevalence of non-communicable diseases and application to dementia in Germany. Theor Popul Biol 2014; 92: 62–8. 24333220 10.1016/j.tpb.2013.11.006

[12] Tönnies T, Röckl S, Hoyer A, Heidemann C, Baumert J, Du Y, et al. Projected number of people with diagnosed type 2 diabetes in Germany in 2040. Diabet Med 2019; 36: 1217–25. 30659656 10.1111/dme.13902

[13] Ito C, Kurth T, Baune BT, Brinks R. Illness-death model as a framework for chronic disease burden projection: application to mental health epidemiology. Front Epidemiol 2022; 2: 903652. 10.3389/fepid.2022.903652 PMC10910899 38455334

[14] Global Burden of Disease Collaborative Network. Global Burden of Disease Study 2019 (GBD 2019) Results, 2020 (http://ghdx.healthdata.org/gbd-results-tool [cited 15 Feb 2022]).

[15] Federal Statistical Office. 15. Koordinierte Bevölkerungsvorausberechnung. Federal Statistical Office (Destatis), 2022 (https://www.destatis.de/DE/Themen/Gesellschaft-Umwelt/Bevoelkerung/Bevoelkerungsvorausberechnung/begleitheft.html [cited 30 Jun 2023]).

[16] Robert Koch Institute. SurvStat@RKI 2.0. RKI (https://survstat.rki.de [cited 17 Feb 2023]).

[17] Doyle AC, Pollack MH. Establishment of remission criteria for anxiety disorders. J Clin Psychiatry 2003; 64(Suppl 15): 40–5. 14658990

[18] Walker ER, McGee RE, Druss BG. Mortality in mental disorders and global disease burden implications: a systematic review and meta-analysis. JAMA Psychiatry 2015; 72: 334–41. 25671328 10.1001/jamapsychiatry.2014.2502 PMC4461039

[19] Carstensen B, Rønn PF, Jørgensen ME. Components of diabetes prevalence in Denmark 1996–2016 and future trends until 2030. BMJ Open Diabetes Res Care 2020; 8: e001064. 10.1136/bmjdrc-2019-001064 PMC7418686 32784246

[20] Varga TV, Bu F, Dissing AS, Elsenburg LK, Bustamante JJH, Matta J, et al. Loneliness, worries, anxiety, and precautionary behaviours in response to the COVID-19 pandemic: a longitudinal analysis of 200,000 western and northern Europeans. Lancet Reg Health Eur 2021; 2: 100020. 33870246 10.1016/j.lanepe.2020.100020 PMC8042675

[21] Jia H, Guerin RJ, Barile JP, Okun AH, McKnight-Eily L, Blumberg SJ, et al. National and state trends in anxiety and depression severity scores among adults during the COVID-19 pandemic – United States, 2020–2021. MMWR Morb Mortal Wkly Rep 2021; 70: 1427–32. 34618798 10.15585/mmwr.mm7040e3 PMC12364538

[22] Tolksdorf K, Loenenbach A, Buda S. Dritte aktualisierung der ‘retrospektiven phaseneinteilung der COVID-19-pandemie in Deutschland’ [Third update of the “retrospective phasing of the COVID-19 pandemic in Germany’]. Epidemiol Bull 2022; 38: 3–6.

[23] Markevich N, Gertner I. Comparison among methods for calculating FWHM. Nucl Instrum Methods Phys Res A 1989; 283: 72–7.

[24] R Core Team. R: A Language and Environment for Statistical Computing. R Foundation for Statistical Computing, 2022 (https://www.R-project.org/).

[25] Posit team. RStudio: Integrated Development Environment for R. 2023 (http://www.posit.co/).

[26] McBain RK, Cantor J, Pera MF, Breslau J, Bravata DM, Whaley CM. Mental health service utilization rates among commercially insured adults in the US during the first year of the COVID-19 pandemic. JAMA Health Forum 2023; 4: e224936. 36607697 10.1001/jamahealthforum.2022.4936 PMC9857246

[27] Anderson KN, Radhakrishnan L, Lane RI, Sheppard M, DeVies J, Azondekon R, et al. Changes and inequities in adult mental health-related emergency department visits during the COVID-19 pandemic in the US. JAMA Psychiatry 2022; 79: 475–85. 35293958 10.1001/jamapsychiatry.2022.0164 PMC8928092

[28] Manchia M, Gathier AW, Yapici-Eser H, Schmidt MV, de Quervain D, van Amelsvoort T, et al. The impact of the prolonged COVID-19 pandemic on stress resilience and mental health: a critical review across waves. Eur Neuropsychopharmacol 2022; 55: 22–83. 34818601 10.1016/j.euroneuro.2021.10.864 PMC8554139

[29] Thom J, Mauz E, Peitz D, Kersjes C, Aichberger M, Baumeister H, et al. Establishing a mental health surveillance in Germany: development of a framework concept and indicator set. J Health Monit 2021; 6: 34–63. 35146320 10.25646/8861 PMC8734140

[30] Meier SM, Mattheisen M, Mors O, Mortensen PB, Laursen TM, Penninx BW. Increased mortality among people with anxiety disorders: total population study. Br J Psychiatry 2016; 209: 216–21. 27388572 10.1192/bjp.bp.115.171975 PMC5082973

[31] Neelam K, Duddu V, Anyim N, Neelam J, Lewis S. Pandemics and pre-existing mental illness: a systematic review and meta-analysis. Brain Behav Immun Health 2021; 10: 100177. 33251527 10.1016/j.bbih.2020.100177 PMC7683956

[32] Blackmore R, Boyle JA, Fazel M, Ranasinha S, Gray KM, Fitzgerald G, et al. The prevalence of mental illness in refugees and asylum seekers: a systematic review and meta-analysis. PLoS Med 2020; 17: e1003337. 32956381 10.1371/journal.pmed.1003337 PMC7505461

[33] Lebano A, Hamed S, Bradby H, Gil-Salmerón A, Durá-Ferrandis E, Garcés-Ferrer J, et al. Migrants’ and refugees’ health status and healthcare in Europe: a scoping literature review. BMC Public Health 2020; 20: 1039. 32605605 10.1186/s12889-020-08749-8 PMC7329528

