# Supplementary material for: Projections of anxiety disorder prevalence during and beyond the COVID-19 pandemic in Germany using the illness–death model
Source: BJPsych Open. 2024 Oct 10;10(5):e174. doi: 10.1192/bjo.2024.754 (PMC11536217; doi:10.1192/bjo.2024.754)
Supplement: Ito et al. supplementary material [file S2056472424007543sup001.docx]

**SUPPLEMENTAL MATERIAL**

**Figure S1.** Anxiety incidence increase during the COVID-19 pandemic waves and model parameters

**Table S1.** Parameters for incidence model for projections: scenarios 0-81

**Figure S2.** Projected number of women with anxiety disorders in Germany from 2019 to 2030

**Figure S3.** Projected number of men with anxiety disorders in Germany from 2019 to 2030

**Table S2.** Projected number and prevalence of anxiety disorders in 2020, 2021, 2023, and 2030 in Germany, by sex and scenario (all 81 scenarios)

**Figure S1.** Anxiety incidence increase during the COVID-19 pandemic waves and model parameters


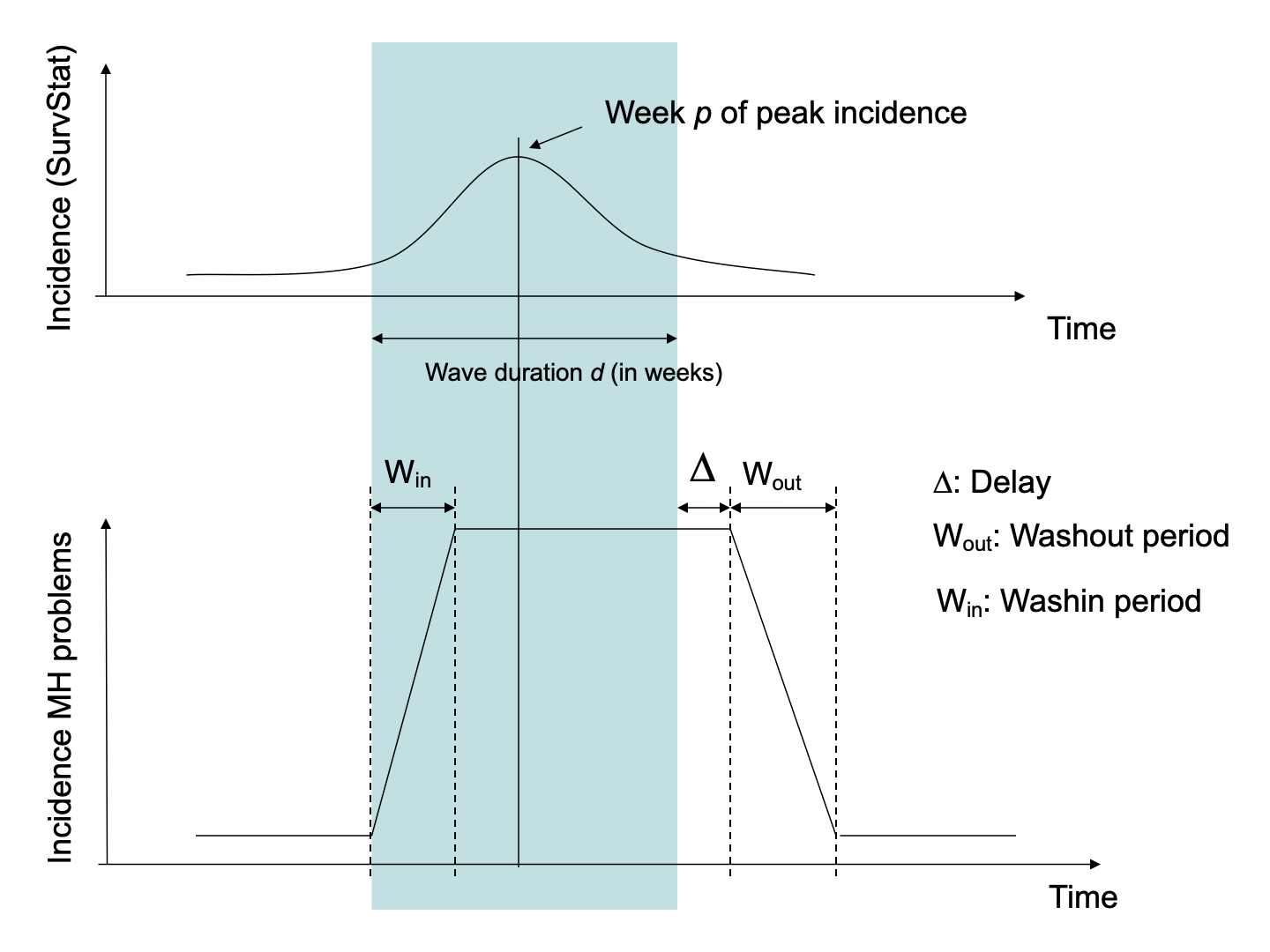


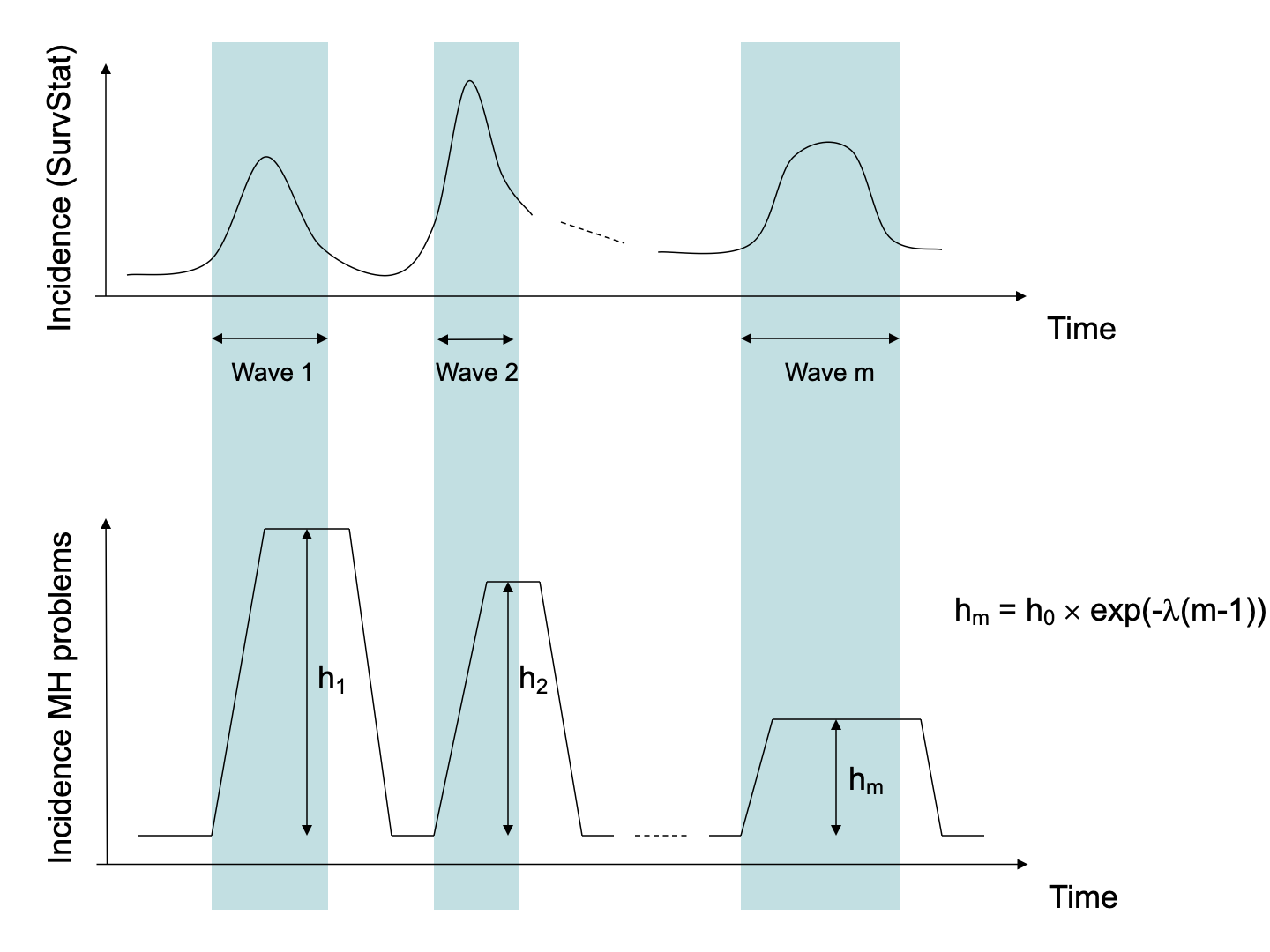
**Table S1.** Parameters for incidence model for projections: scenarios 0-81

| Scenario | Wash-in (*W_in_*) | Wash-out (*W_out_*) | Delay (*Δ*) | Decay (*λ*) | Incidence increase (*h_0_*) |
| --- | --- | --- | --- | --- | --- |
| 0*^1^* | 0.00 | 0.00 | 0.00 | 0.00 | 0 |
| 1 | 0.10 | 0.10 | 0.00 | 0.10 | 1 |
| 2 | 0.20 | 0.20 | 0.00 | 0.10 | 1 |
| 3 | 0.30 | 0.30 | 0.00 | 0.10 | 1 |
| 4 | 0.10 | 0.10 | 0.25 | 0.10 | 1 |
| 5 | 0.20 | 0.20 | 0.25 | 0.10 | 1 |
| 6 | 0.30 | 0.30 | 0.25 | 0.10 | 1 |
| 7 | 0.10 | 0.10 | 0.50 | 0.10 | 1 |
| 8 | 0.20 | 0.20 | 0.50 | 0.10 | 1 |
| 9 | 0.30 | 0.30 | 0.50 | 0.10 | 1 |
| 10 | 0.10 | 0.10 | 0.00 | 0.20 | 1 |
| 11 | 0.20 | 0.20 | 0.00 | 0.20 | 1 |
| 12 | 0.30 | 0.30 | 0.00 | 0.20 | 1 |
| 13 | 0.10 | 0.10 | 0.25 | 0.20 | 1 |
| 14 | 0.20 | 0.20 | 0.25 | 0.20 | 1 |
| 15 | 0.30 | 0.30 | 0.25 | 0.20 | 1 |
| 16 | 0.10 | 0.10 | 0.50 | 0.20 | 1 |
| 17 | 0.20 | 0.20 | 0.50 | 0.20 | 1 |
| 18 | 0.30 | 0.30 | 0.50 | 0.20 | 1 |
| 19 | 0.10 | 0.10 | 0.00 | 0.30 | 1 |
| 20 | 0.20 | 0.20 | 0.00 | 0.30 | 1 |
| 21 | 0.30 | 0.30 | 0.00 | 0.30 | 1 |
| 22 | 0.10 | 0.10 | 0.25 | 0.30 | 1 |
| 23 | 0.20 | 0.20 | 0.25 | 0.30 | 1 |
| 24 | 0.30 | 0.30 | 0.25 | 0.30 | 1 |
| 25 | 0.10 | 0.10 | 0.50 | 0.30 | 1 |
| 26 | 0.20 | 0.20 | 0.50 | 0.30 | 1 |
| 27 | 0.30 | 0.30 | 0.50 | 0.30 | 1 |
| 28 | 0.10 | 0.10 | 0.00 | 0.10 | 5 |
| 29 | 0.20 | 0.20 | 0.00 | 0.10 | 5 |
| 30 | 0.30 | 0.30 | 0.00 | 0.10 | 5 |
| 31 | 0.10 | 0.10 | 0.25 | 0.10 | 5 |
| 32 | 0.20 | 0.20 | 0.25 | 0.10 | 5 |
| 33 | 0.30 | 0.30 | 0.25 | 0.10 | 5 |
| 34 | 0.10 | 0.10 | 0.50 | 0.10 | 5 |
| 35 | 0.20 | 0.20 | 0.50 | 0.10 | 5 |
| 36 | 0.30 | 0.30 | 0.50 | 0.10 | 5 |
| 37 | 0.10 | 0.10 | 0.00 | 0.20 | 5 |
| 38 | 0.20 | 0.20 | 0.00 | 0.20 | 5 |
| 39 | 0.30 | 0.30 | 0.00 | 0.20 | 5 |
| 40 | 0.10 | 0.10 | 0.25 | 0.20 | 5 |
| 41 | 0.20 | 0.20 | 0.25 | 0.20 | 5 |
| 42 | 0.30 | 0.30 | 0.25 | 0.20 | 5 |
| 43 | 0.10 | 0.10 | 0.50 | 0.20 | 5 |
| 44 | 0.20 | 0.20 | 0.50 | 0.20 | 5 |
| 45 | 0.30 | 0.30 | 0.50 | 0.20 | 5 |
| 46 | 0.10 | 0.10 | 0.00 | 0.30 | 5 |
| 47 | 0.20 | 0.20 | 0.00 | 0.30 | 5 |
| 48 | 0.30 | 0.30 | 0.00 | 0.30 | 5 |
| 49 | 0.10 | 0.10 | 0.25 | 0.30 | 5 |
| 50 | 0.20 | 0.20 | 0.25 | 0.30 | 5 |
| 51 | 0.30 | 0.30 | 0.25 | 0.30 | 5 |
| 52 | 0.10 | 0.10 | 0.50 | 0.30 | 5 |
| 53 | 0.20 | 0.20 | 0.50 | 0.30 | 5 |
| 54 | 0.30 | 0.30 | 0.50 | 0.30 | 5 |
| 55 | 0.10 | 0.10 | 0.00 | 0.10 | 10 |
| 56 | 0.20 | 0.20 | 0.00 | 0.10 | 10 |
| 57 | 0.30 | 0.30 | 0.00 | 0.10 | 10 |
| 58 | 0.10 | 0.10 | 0.25 | 0.10 | 10 |
| 59 | 0.20 | 0.20 | 0.25 | 0.10 | 10 |
| 60 | 0.30 | 0.30 | 0.25 | 0.10 | 10 |
| 61 | 0.10 | 0.10 | 0.50 | 0.10 | 10 |
| 62 | 0.20 | 0.20 | 0.50 | 0.10 | 10 |
| 63 | 0.30 | 0.30 | 0.50 | 0.10 | 10 |
| 64 | 0.10 | 0.10 | 0.00 | 0.20 | 10 |
| 65 | 0.20 | 0.20 | 0.00 | 0.20 | 10 |
| 66 | 0.30 | 0.30 | 0.00 | 0.20 | 10 |
| 67 | 0.10 | 0.10 | 0.25 | 0.20 | 10 |
| 68 | 0.20 | 0.20 | 0.25 | 0.20 | 10 |
| 69 | 0.30 | 0.30 | 0.25 | 0.20 | 10 |
| 70 | 0.10 | 0.10 | 0.50 | 0.20 | 10 |
| 71 | 0.20 | 0.20 | 0.50 | 0.20 | 10 |
| 72 | 0.30 | 0.30 | 0.50 | 0.20 | 10 |
| 73 | 0.10 | 0.10 | 0.00 | 0.30 | 10 |
| 74 | 0.20 | 0.20 | 0.00 | 0.30 | 10 |
| 75 | 0.30 | 0.30 | 0.00 | 0.30 | 10 |
| 76 | 0.10 | 0.10 | 0.25 | 0.30 | 10 |
| 77 | 0.20 | 0.20 | 0.25 | 0.30 | 10 |
| 78 | 0.30 | 0.30 | 0.25 | 0.30 | 10 |
| 79 | 0.10 | 0.10 | 0.50 | 0.30 | 10 |
| 80 | 0.20 | 0.20 | 0.50 | 0.30 | 10 |
| 81 | 0.30 | 0.30 | 0.50 | 0.30 | 10 |
| *W_in_*: wash-in period, a period from the start of a given pandemic wave to the time when the anxiety disorder incidence peaks during the wave.  *W_out_*: wash-out period, a time between the moment the elevated anxiety disorder incidence starts to decline and the moment when the incidence is no longer raised higher than what would be expected from the historical trends.  *Δ*: delay, during which the COVID-19 incidence starts to subside while the anxiety disorder incidence remains elevated.  *h_0_*: the magnitude of an anxiety disorder incidence increase.  *λ*: decay constant at which *h_0_* gradually diminishes from one wave to another.  *^1^* Scenario 0 is the baseline projection where the COVID-19 pandemic is assumed to have no effects on anxiety disorder incidence. | | | | | |

**Figure S2.** Projected number of women with anxiety disorders in Germany from 2019 to 2030


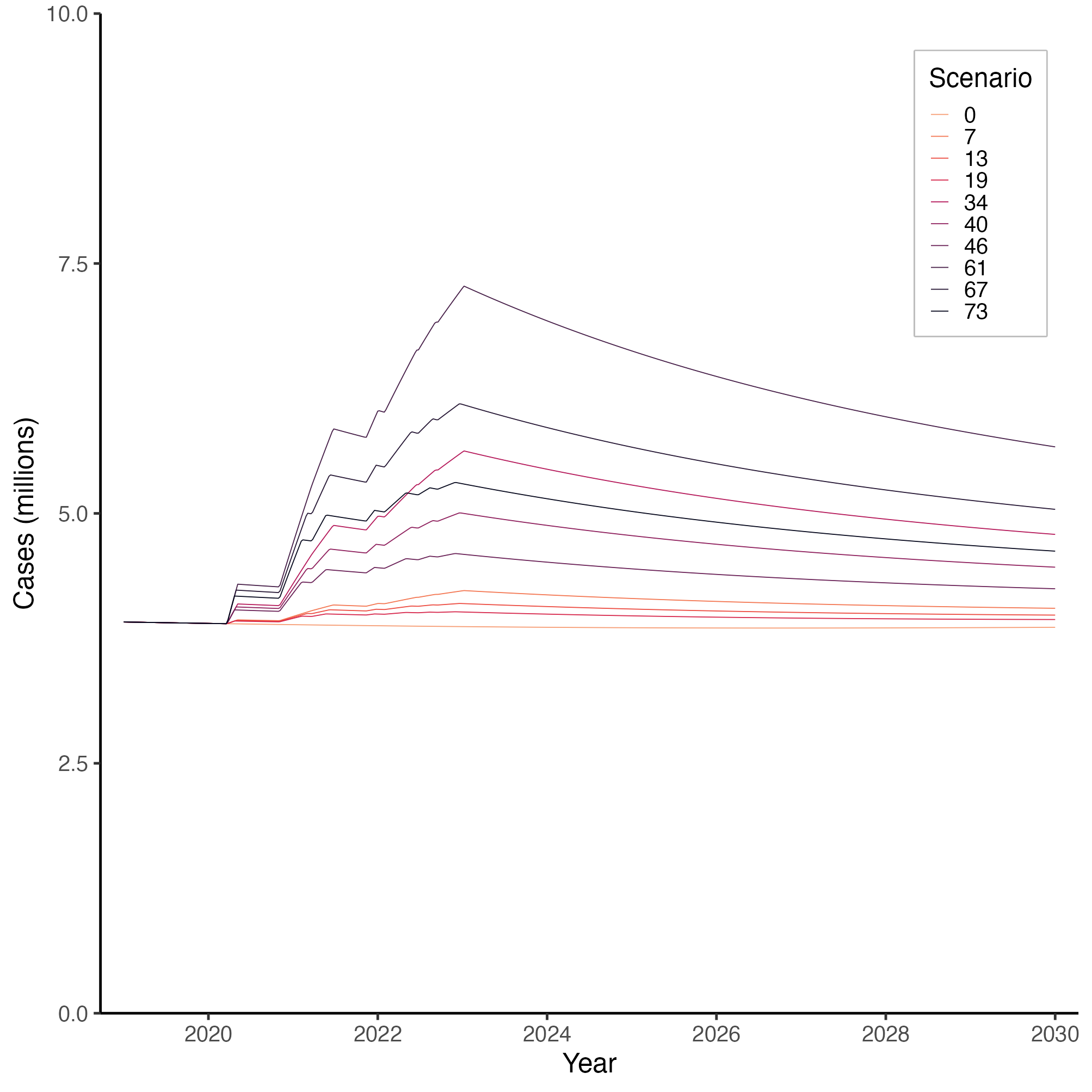


Number of women (millions) with anxiety disorders in Germany from 2019 to 2030, projected with the illness-death model under 10 selected scenarios.

In Scenarios 7, 13, and 19, the incidence increase *h_0_* was set to 1. Scenario 7 assumed a large delay and a small decay constant (*Δ* = 0.5; *λ* = 0.1); Scenario 13 mimicked a moderate delay and decay (*Δ* = 0.25, *λ* = 0.2); and Scenario 19 simulated no delay and a large decay constant (*Δ* = 0, *λ* = 0.3).

In Scenarios 34, 40, and 46, *h_0_* was set to 5. We provided Scenario 34 with a large delay and a small decay constant (*Δ* = 0.5; *λ* = 0.1), Scenario 13 with a moderate delay and decay (*Δ* = 0.25, *λ* = 0.2), and Scenario 19 with no delay and a large decay (*Δ* = 0, *λ* = 0.3).

In Scenarios 61, 67, and 73, *h_0_* was set to 10. We provided Scenario 61 with a large delay and a small decay constant (*Δ* = 0.5; *λ* = 0.1), Scenario 67 with a moderate delay and decay (*Δ* = 0.25, *λ* = 0.2), and Scenario 73 with no delay and a large decay (*Δ* = 0, *λ* = 0.3).

**Figure S3.** Projected number of men with anxiety disorders in Germany from 2019 to 2030


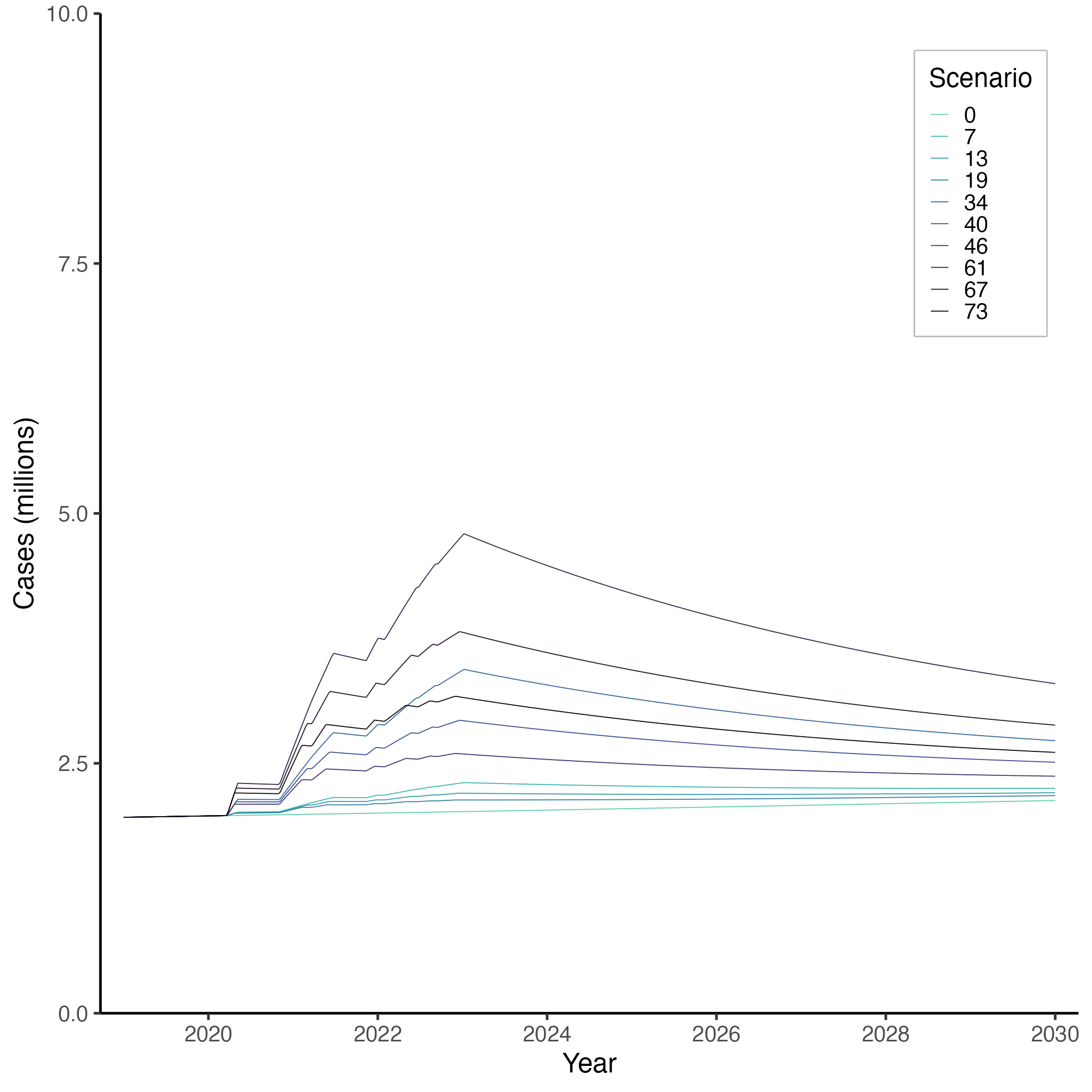


Number of men (millions) with anxiety disorders in Germany from 2019 to 2030, projected with the illness-death model under 10 selected scenarios.

In Scenarios 7, 13, and 19, the incidence increase *h_0_* was set to 1. Scenario 7 assumed a large delay and a small decay constant (*Δ* = 0.5; *λ* = 0.1); Scenario 13 mimicked a moderate delay and decay (*Δ* = 0.25, *λ* = 0.2); and Scenario 19 simulated no delay and a large decay constant (*Δ* = 0, *λ* = 0.3).

In Scenarios 34, 40, and 46, *h_0_* was set to 5. We provided Scenario 34 with a large delay and a small decay constant (*Δ* = 0.5; *λ* = 0.1), Scenario 13 with a moderate delay and decay (*Δ* = 0.25, *λ* = 0.2), and Scenario 19 with no delay and a large decay (*Δ* = 0, *λ* = 0.3).

In Scenarios 61, 67, and 73, *h_0_* was set to 10. We provided Scenario 61 with a large delay and a small decay constant (*Δ* = 0.5; *λ* = 0.1), Scenario 67 with a moderate delay and decay (*Δ* = 0.25, *λ* = 0.2), and Scenario 73 with no delay and a large decay (*Δ* = 0, *λ* = 0.3).

**Table S2.** Projected number and prevalence of anxiety disorders in 2020, 2021, 2023, and 2030 in Germany, by sex and scenario (all 81 scenarios)

| Estimated number and prevalence of anxiety disorders in Germany by sex | | | | | | | | | | | | | |
| --- | --- | --- | --- | --- | --- | --- | --- | --- | --- | --- | --- | --- | --- |
|  |  |  |  |  |  | 2020 | | 2021 | | 2023 | | 2030 | |
| Scenario | *W_in_* | *W_out_* | *Δ* | *λ* | *h_0_* | Case (millions) | Prevalence (%) | Case (millions) | Prevalence (%) | Case (millions) | Prevalence (%) | Case (millions) | Prevalence (%) |
| Women | | | | | | | | | | | | | |
| 0 | 0.00 | 0.00 | 0.00 | 0.00 | 0.00 | 3.900 | 9.47 | 3.887 | 9.49 | 3.867 | 9.56% | 3.860 | 9.96 |
| 1 | 0.10 | 0.10 | 0.00 | 0.10 | 1.00 | 3.900 | 9.47 | 3.958 | 9.67 | 4.112 | 10.17% | 3.989 | 10.30 |
| 2 | 0.20 | 0.20 | 0.00 | 0.10 | 1.00 | 3.900 | 9.47 | 3.953 | 9.66 | 4.111 | 10.17% | 3.989 | 10.29 |
| 3 | 0.30 | 0.30 | 0.00 | 0.10 | 1.00 | 3.900 | 9.47 | 3.950 | 9.65 | 4.112 | 10.17% | 3.989 | 10.30 |
| 4 | 0.10 | 0.10 | 0.25 | 0.10 | 1.00 | 3.900 | 9.47 | 3.964 | 9.68 | 4.171 | 10.30% | 4.021 | 10.37 |
| 5 | 0.20 | 0.20 | 0.25 | 0.10 | 1.00 | 3.900 | 9.47 | 3.959 | 9.67 | 4.172 | 10.30% | 4.021 | 10.37 |
| 6 | 0.30 | 0.30 | 0.25 | 0.10 | 1.00 | 3.900 | 9.47 | 3.956 | 9.66 | 4.172 | 10.30% | 4.021 | 10.37 |
| 7 | 0.10 | 0.10 | 0.50 | 0.10 | 1.00 | 3.900 | 9.47 | 3.969 | 9.69 | 4.226 | 10.42% | 4.051 | 10.44 |
| 8 | 0.20 | 0.20 | 0.50 | 0.10 | 1.00 | 3.900 | 9.47 | 3.966 | 9.69 | 4.221 | 10.41% | 4.049 | 10.43 |
| 9 | 0.30 | 0.30 | 0.50 | 0.10 | 1.00 | 3.900 | 9.47 | 3.962 | 9.68 | 4.215 | 10.40% | 4.047 | 10.43 |
| 10 | 0.10 | 0.10 | 0.00 | 0.20 | 1.00 | 3.900 | 9.47 | 3.954 | 9.66 | 4.053 | 10.04% | 3.959 | 10.23 |
| 11 | 0.20 | 0.20 | 0.00 | 0.20 | 1.00 | 3.900 | 9.47 | 3.949 | 9.65 | 4.052 | 10.04% | 3.958 | 10.22 |
| 12 | 0.30 | 0.30 | 0.00 | 0.20 | 1.00 | 3.900 | 9.47 | 3.947 | 9.64 | 4.053 | 10.04% | 3.959 | 10.22 |
| 13 | 0.10 | 0.10 | 0.25 | 0.20 | 1.00 | 3.900 | 9.47 | 3.959 | 9.67 | 4.098 | 10.14% | 3.983 | 10.28 |
| 14 | 0.20 | 0.20 | 0.25 | 0.20 | 1.00 | 3.900 | 9.47 | 3.955 | 9.66 | 4.099 | 10.14% | 3.983 | 10.28 |
| 15 | 0.30 | 0.30 | 0.25 | 0.20 | 1.00 | 3.900 | 9.47 | 3.953 | 9.66 | 4.099 | 10.14% | 3.983 | 10.28 |
| 16 | 0.10 | 0.10 | 0.50 | 0.20 | 1.00 | 3.900 | 9.47 | 3.965 | 9.68 | 4.140 | 10.23% | 4.006 | 10.33 |
| 17 | 0.20 | 0.20 | 0.50 | 0.20 | 1.00 | 3.900 | 9.47 | 3.962 | 9.68 | 4.137 | 10.22% | 4.004 | 10.33 |
| 18 | 0.30 | 0.30 | 0.50 | 0.20 | 1.00 | 3.900 | 9.47 | 3.959 | 9.67 | 4.133 | 10.21% | 4.003 | 10.33 |
| 19 | 0.10 | 0.10 | 0.00 | 0.30 | 1.00 | 3.900 | 9.47 | 3.950 | 9.65 | 4.014 | 9.95% | 3.938 | 10.18 |
| 20 | 0.20 | 0.20 | 0.00 | 0.30 | 1.00 | 3.900 | 9.47 | 3.945 | 9.64 | 4.013 | 9.95% | 3.938 | 10.18 |
| 21 | 0.30 | 0.30 | 0.00 | 0.30 | 1.00 | 3.900 | 9.47 | 3.943 | 9.64 | 4.013 | 9.95% | 3.938 | 10.18 |
| 22 | 0.10 | 0.10 | 0.25 | 0.30 | 1.00 | 3.900 | 9.47 | 3.956 | 9.66 | 4.049 | 10.03% | 3.957 | 10.22 |
| 23 | 0.20 | 0.20 | 0.25 | 0.30 | 1.00 | 3.900 | 9.47 | 3.952 | 9.65 | 4.049 | 10.03% | 3.957 | 10.22 |
| 24 | 0.30 | 0.30 | 0.25 | 0.30 | 1.00 | 3.900 | 9.47 | 3.949 | 9.65 | 4.049 | 10.03% | 3.957 | 10.22 |
| 25 | 0.10 | 0.10 | 0.50 | 0.30 | 1.00 | 3.900 | 9.47 | 3.961 | 9.68 | 4.082 | 10.10% | 3.975 | 10.26 |
| 26 | 0.20 | 0.20 | 0.50 | 0.30 | 1.00 | 3.900 | 9.47 | 3.958 | 9.67 | 4.080 | 10.10% | 3.974 | 10.26 |
| 27 | 0.30 | 0.30 | 0.50 | 0.30 | 1.00 | 3.900 | 9.47 | 3.955 | 9.66 | 4.077 | 10.09% | 3.973 | 10.26 |
| 28 | 0.10 | 0.10 | 0.00 | 0.10 | 5.00 | 3.900 | 9.47 | 4.242 | 10.29 | 5.069 | 12.24% | 4.495 | 11.45 |
| 29 | 0.20 | 0.20 | 0.00 | 0.10 | 5.00 | 3.900 | 9.47 | 4.216 | 10.23 | 5.064 | 12.23% | 4.492 | 11.44 |
| 30 | 0.30 | 0.30 | 0.00 | 0.10 | 5.00 | 3.900 | 9.47 | 4.202 | 10.20 | 5.069 | 12.24% | 4.495 | 11.45 |
| 31 | 0.10 | 0.10 | 0.25 | 0.10 | 5.00 | 3.900 | 9.47 | 4.270 | 10.35 | 5.353 | 12.84% | 4.645 | 11.79 |
| 32 | 0.20 | 0.20 | 0.25 | 0.10 | 5.00 | 3.900 | 9.47 | 4.247 | 10.30 | 5.358 | 12.85% | 4.647 | 11.79 |
| 33 | 0.30 | 0.30 | 0.25 | 0.10 | 5.00 | 3.900 | 9.47 | 4.232 | 10.27 | 5.356 | 12.85% | 4.647 | 11.79 |
| 34 | 0.10 | 0.10 | 0.50 | 0.10 | 5.00 | 3.900 | 9.47 | 4.297 | 10.41 | 5.614 | 13.39% | 4.790 | 12.11 |
| 35 | 0.20 | 0.20 | 0.50 | 0.10 | 5.00 | 3.900 | 9.47 | 4.279 | 10.37 | 5.590 | 13.34% | 4.782 | 12.09 |
| 36 | 0.30 | 0.30 | 0.50 | 0.10 | 5.00 | 3.900 | 9.47 | 4.263 | 10.34 | 5.562 | 13.28% | 4.771 | 12.07 |
| 37 | 0.10 | 0.10 | 0.00 | 0.20 | 5.00 | 3.900 | 9.47 | 4.220 | 10.24 | 4.784 | 11.64% | 4.347 | 11.12 |
| 38 | 0.20 | 0.20 | 0.00 | 0.20 | 5.00 | 3.900 | 9.47 | 4.196 | 10.19 | 4.779 | 11.63% | 4.344 | 11.11 |
| 39 | 0.30 | 0.30 | 0.00 | 0.20 | 5.00 | 3.900 | 9.47 | 4.184 | 10.16 | 4.784 | 11.64% | 4.346 | 11.11 |
| 40 | 0.10 | 0.10 | 0.25 | 0.20 | 5.00 | 3.900 | 9.47 | 4.248 | 10.30 | 5.002 | 12.10% | 4.462 | 11.38 |
| 41 | 0.20 | 0.20 | 0.25 | 0.20 | 5.00 | 3.900 | 9.47 | 4.228 | 10.26 | 5.005 | 12.11% | 4.463 | 11.38 |
| 42 | 0.30 | 0.30 | 0.25 | 0.20 | 5.00 | 3.900 | 9.47 | 4.214 | 10.23 | 5.004 | 12.11% | 4.463 | 11.38 |
| 43 | 0.10 | 0.10 | 0.50 | 0.20 | 5.00 | 3.900 | 9.47 | 4.276 | 10.36 | 5.204 | 12.53% | 4.573 | 11.63 |
| 44 | 0.20 | 0.20 | 0.50 | 0.20 | 5.00 | 3.900 | 9.47 | 4.259 | 10.33 | 5.188 | 12.50% | 4.567 | 11.61 |
| 45 | 0.30 | 0.30 | 0.50 | 0.20 | 5.00 | 3.900 | 9.47 | 4.245 | 10.30 | 5.168 | 12.46% | 4.559 | 11.60 |
| 46 | 0.10 | 0.10 | 0.00 | 0.30 | 5.00 | 3.900 | 9.47 | 4.201 | 10.20 | 4.591 | 11.22% | 4.246 | 10.89 |
| 47 | 0.20 | 0.20 | 0.00 | 0.30 | 5.00 | 3.900 | 9.47 | 4.179 | 10.15 | 4.586 | 11.21% | 4.243 | 10.88 |
| 48 | 0.30 | 0.30 | 0.00 | 0.30 | 5.00 | 3.900 | 9.47 | 4.168 | 10.13 | 4.590 | 11.22% | 4.245 | 10.88 |
| 49 | 0.10 | 0.10 | 0.25 | 0.30 | 5.00 | 3.900 | 9.47 | 4.229 | 10.26 | 4.763 | 11.59% | 4.337 | 11.09 |
| 50 | 0.20 | 0.20 | 0.25 | 0.30 | 5.00 | 3.900 | 9.47 | 4.210 | 10.22 | 4.765 | 11.60% | 4.338 | 11.10 |
| 51 | 0.30 | 0.30 | 0.25 | 0.30 | 5.00 | 3.900 | 9.47 | 4.198 | 10.20 | 4.765 | 11.60% | 4.338 | 11.10 |
| 52 | 0.10 | 0.10 | 0.50 | 0.30 | 5.00 | 3.900 | 9.47 | 4.257 | 10.32 | 4.925 | 11.94% | 4.425 | 11.29 |
| 53 | 0.20 | 0.20 | 0.50 | 0.30 | 5.00 | 3.900 | 9.47 | 4.241 | 10.29 | 4.914 | 11.91% | 4.421 | 11.28 |
| 54 | 0.30 | 0.30 | 0.50 | 0.30 | 5.00 | 3.900 | 9.47 | 4.229 | 10.26 | 4.900 | 11.88% | 4.415 | 11.27 |
| 55 | 0.10 | 0.10 | 0.00 | 0.10 | 10.00 | 3.900 | 9.47 | 4.592 | 11.05 | 6.220 | 14.62% | 5.105 | 12.81 |
| 56 | 0.20 | 0.20 | 0.00 | 0.10 | 10.00 | 3.900 | 9.47 | 4.542 | 10.94 | 6.210 | 14.60% | 5.099 | 12.79 |
| 57 | 0.30 | 0.30 | 0.00 | 0.10 | 10.00 | 3.900 | 9.47 | 4.514 | 10.88 | 6.220 | 14.62% | 5.104 | 12.80 |
| 58 | 0.10 | 0.10 | 0.25 | 0.10 | 10.00 | 3.900 | 9.47 | 4.648 | 11.16 | 6.761 | 15.69% | 5.391 | 13.43 |
| 59 | 0.20 | 0.20 | 0.25 | 0.10 | 10.00 | 3.900 | 9.47 | 4.604 | 11.07 | 6.771 | 15.71% | 5.396 | 13.44 |
| 60 | 0.30 | 0.30 | 0.25 | 0.10 | 10.00 | 3.900 | 9.47 | 4.574 | 11.01 | 6.767 | 15.70% | 5.395 | 13.44 |
| 61 | 0.10 | 0.10 | 0.50 | 0.10 | 10.00 | 3.900 | 9.47 | 4.703 | 11.28 | 7.254 | 16.64% | 5.665 | 14.02 |
| 62 | 0.20 | 0.20 | 0.50 | 0.10 | 10.00 | 3.900 | 9.47 | 4.666 | 11.20 | 7.209 | 16.56% | 5.650 | 13.98 |
| 63 | 0.30 | 0.30 | 0.50 | 0.10 | 10.00 | 3.900 | 9.47 | 4.634 | 11.14 | 7.156 | 16.46% | 5.630 | 13.94 |
| 64 | 0.10 | 0.10 | 0.00 | 0.20 | 10.00 | 3.900 | 9.47 | 4.550 | 10.96 | 5.671 | 13.50% | 4.819 | 12.18 |
| 65 | 0.20 | 0.20 | 0.00 | 0.20 | 10.00 | 3.900 | 9.47 | 4.503 | 10.86 | 5.661 | 13.48% | 4.812 | 12.16 |
| 66 | 0.30 | 0.30 | 0.00 | 0.20 | 10.00 | 3.900 | 9.47 | 4.479 | 10.80 | 5.670 | 13.50% | 4.817 | 12.17 |
| 67 | 0.10 | 0.10 | 0.25 | 0.20 | 10.00 | 3.900 | 9.47 | 4.605 | 11.07 | 6.090 | 14.36% | 5.041 | 12.66 |
| 68 | 0.20 | 0.20 | 0.25 | 0.20 | 10.00 | 3.900 | 9.47 | 4.565 | 10.99 | 6.096 | 14.37% | 5.043 | 12.67 |
| 69 | 0.30 | 0.30 | 0.25 | 0.20 | 10.00 | 3.900 | 9.47 | 4.539 | 10.93 | 6.095 | 14.37% | 5.044 | 12.67 |
| 70 | 0.10 | 0.10 | 0.50 | 0.20 | 10.00 | 3.900 | 9.47 | 4.660 | 11.19 | 6.477 | 15.13% | 5.254 | 13.13 |
| 71 | 0.20 | 0.20 | 0.50 | 0.20 | 10.00 | 3.900 | 9.47 | 4.627 | 11.12 | 6.446 | 15.07% | 5.242 | 13.10 |
| 72 | 0.30 | 0.30 | 0.50 | 0.20 | 10.00 | 3.900 | 9.47 | 4.598 | 11.06 | 6.409 | 15.00% | 5.227 | 13.07 |
| 73 | 0.10 | 0.10 | 0.00 | 0.30 | 10.00 | 3.900 | 9.47 | 4.512 | 10.87 | 5.296 | 12.72% | 4.622 | 11.74 |
| 74 | 0.20 | 0.20 | 0.00 | 0.30 | 10.00 | 3.900 | 9.47 | 4.468 | 10.78 | 5.285 | 12.70% | 4.616 | 11.72 |
| 75 | 0.30 | 0.30 | 0.00 | 0.30 | 10.00 | 3.900 | 9.47 | 4.447 | 10.73 | 5.294 | 12.72% | 4.620 | 11.73 |
| 76 | 0.10 | 0.10 | 0.25 | 0.30 | 10.00 | 3.900 | 9.47 | 4.567 | 10.99 | 5.630 | 13.42% | 4.800 | 12.13 |
| 77 | 0.20 | 0.20 | 0.25 | 0.30 | 10.00 | 3.900 | 9.47 | 4.530 | 10.91 | 5.633 | 13.42% | 4.801 | 12.14 |
| 78 | 0.30 | 0.30 | 0.25 | 0.30 | 10.00 | 3.900 | 9.47 | 4.507 | 10.86 | 5.633 | 13.42% | 4.802 | 12.14 |
| 79 | 0.10 | 0.10 | 0.50 | 0.30 | 10.00 | 3.900 | 9.47 | 4.622 | 11.11 | 5.942 | 14.06% | 4.970 | 12.51 |
| 80 | 0.20 | 0.20 | 0.50 | 0.30 | 10.00 | 3.900 | 9.47 | 4.592 | 11.05 | 5.920 | 14.01% | 4.961 | 12.49 |
| 81 | 0.30 | 0.30 | 0.50 | 0.30 | 10.00 | 3.900 | 9.47 | 4.567 | 10.99 | 5.893 | 13.96% | 4.949 | 12.46 |
| Men | | | | | | | | | | | | | |
| 0 | 0.00 | 0.00 | 0.00 | 0.00 | 0.00 | 1.974 | 4.89 | 1.988 | 4.93 | 2.017 | 5.02 | 2.128 | 5.40 |
| 1 | 0.10 | 0.10 | 0.00 | 0.10 | 1.00 | 1.974 | 4.89 | 2.045 | 5.07 | 2.213 | 5.51 | 2.211 | 5.61 |
| 2 | 0.20 | 0.20 | 0.00 | 0.10 | 1.00 | 1.974 | 4.89 | 2.041 | 5.06 | 2.212 | 5.50 | 2.210 | 5.60 |
| 3 | 0.30 | 0.30 | 0.00 | 0.10 | 1.00 | 1.974 | 4.89 | 2.039 | 5.06 | 2.213 | 5.51 | 2.211 | 5.61 |
| 4 | 0.10 | 0.10 | 0.25 | 0.10 | 1.00 | 1.974 | 4.89 | 2.050 | 5.08 | 2.261 | 5.62 | 2.231 | 5.65 |
| 5 | 0.20 | 0.20 | 0.25 | 0.10 | 1.00 | 1.974 | 4.89 | 2.046 | 5.08 | 2.262 | 5.62 | 2.231 | 5.65 |
| 6 | 0.30 | 0.30 | 0.25 | 0.10 | 1.00 | 1.974 | 4.89 | 2.044 | 5.07 | 2.261 | 5.62 | 2.231 | 5.65 |
| 7 | 0.10 | 0.10 | 0.50 | 0.10 | 1.00 | 1.974 | 4.89 | 2.054 | 5.09 | 2.305 | 5.72 | 2.250 | 5.70 |
| 8 | 0.20 | 0.20 | 0.50 | 0.10 | 1.00 | 1.974 | 4.89 | 2.051 | 5.09 | 2.301 | 5.71 | 2.249 | 5.70 |
| 9 | 0.30 | 0.30 | 0.50 | 0.10 | 1.00 | 1.974 | 4.89 | 2.049 | 5.08 | 2.296 | 5.70 | 2.248 | 5.69 |
| 10 | 0.10 | 0.10 | 0.00 | 0.20 | 1.00 | 1.974 | 4.89 | 2.042 | 5.06 | 2.165 | 5.39 | 2.191 | 5.56 |
| 11 | 0.20 | 0.20 | 0.00 | 0.20 | 1.00 | 1.974 | 4.89 | 2.038 | 5.06 | 2.164 | 5.39 | 2.190 | 5.56 |
| 12 | 0.30 | 0.30 | 0.00 | 0.20 | 1.00 | 1.974 | 4.89 | 2.036 | 5.05 | 2.165 | 5.39 | 2.191 | 5.56 |
| 13 | 0.10 | 0.10 | 0.25 | 0.20 | 1.00 | 1.974 | 4.89 | 2.046 | 5.08 | 2.201 | 5.48 | 2.206 | 5.59 |
| 14 | 0.20 | 0.20 | 0.25 | 0.20 | 1.00 | 1.974 | 4.89 | 2.043 | 5.07 | 2.202 | 5.48 | 2.206 | 5.59 |
| 15 | 0.30 | 0.30 | 0.25 | 0.20 | 1.00 | 1.974 | 4.89 | 2.041 | 5.06 | 2.202 | 5.48 | 2.206 | 5.59 |
| 16 | 0.10 | 0.10 | 0.50 | 0.20 | 1.00 | 1.974 | 4.89 | 2.051 | 5.09 | 2.235 | 5.56 | 2.220 | 5.63 |
| 17 | 0.20 | 0.20 | 0.50 | 0.20 | 1.00 | 1.974 | 4.89 | 2.048 | 5.08 | 2.232 | 5.55 | 2.220 | 5.63 |
| 18 | 0.30 | 0.30 | 0.50 | 0.20 | 1.00 | 1.974 | 4.89 | 2.046 | 5.07 | 2.229 | 5.54 | 2.219 | 5.62 |
| 19 | 0.10 | 0.10 | 0.00 | 0.30 | 1.00 | 1.974 | 4.89 | 2.039 | 5.06 | 2.133 | 5.32 | 2.177 | 5.52 |
| 20 | 0.20 | 0.20 | 0.00 | 0.30 | 1.00 | 1.974 | 4.89 | 2.035 | 5.05 | 2.132 | 5.32 | 2.177 | 5.52 |
| 21 | 0.30 | 0.30 | 0.00 | 0.30 | 1.00 | 1.974 | 4.89 | 2.033 | 5.04 | 2.133 | 5.32 | 2.177 | 5.52 |
| 22 | 0.10 | 0.10 | 0.25 | 0.30 | 1.00 | 1.974 | 4.89 | 2.043 | 5.07 | 2.161 | 5.38 | 2.189 | 5.55 |
| 23 | 0.20 | 0.20 | 0.25 | 0.30 | 1.00 | 1.974 | 4.89 | 2.040 | 5.06 | 2.162 | 5.39 | 2.189 | 5.55 |
| 24 | 0.30 | 0.30 | 0.25 | 0.30 | 1.00 | 1.974 | 4.89 | 2.038 | 5.06 | 2.162 | 5.39 | 2.189 | 5.55 |
| 25 | 0.10 | 0.10 | 0.50 | 0.30 | 1.00 | 1.974 | 4.89 | 2.048 | 5.08 | 2.188 | 5.45 | 2.200 | 5.58 |
| 26 | 0.20 | 0.20 | 0.50 | 0.30 | 1.00 | 1.974 | 4.89 | 2.045 | 5.07 | 2.186 | 5.44 | 2.200 | 5.58 |
| 27 | 0.30 | 0.30 | 0.50 | 0.30 | 1.00 | 1.974 | 4.89 | 2.043 | 5.07 | 2.184 | 5.44 | 2.199 | 5.58 |
| 28 | 0.10 | 0.10 | 0.00 | 0.10 | 5.00 | 1.974 | 4.89 | 2.275 | 5.61 | 2.985 | 7.29 | 2.535 | 6.37 |
| 29 | 0.20 | 0.20 | 0.00 | 0.10 | 5.00 | 1.974 | 4.89 | 2.255 | 5.56 | 2.982 | 7.28 | 2.533 | 6.37 |
| 30 | 0.30 | 0.30 | 0.00 | 0.10 | 5.00 | 1.974 | 4.89 | 2.243 | 5.54 | 2.986 | 7.29 | 2.535 | 6.38 |
| 31 | 0.10 | 0.10 | 0.25 | 0.10 | 5.00 | 1.974 | 4.89 | 2.298 | 5.66 | 3.217 | 7.81 | 2.632 | 6.60 |
| 32 | 0.20 | 0.20 | 0.25 | 0.10 | 5.00 | 1.974 | 4.89 | 2.280 | 5.62 | 3.222 | 7.82 | 2.634 | 6.61 |
| 33 | 0.30 | 0.30 | 0.25 | 0.10 | 5.00 | 1.974 | 4.89 | 2.268 | 5.59 | 3.220 | 7.82 | 2.634 | 6.61 |
| 34 | 0.10 | 0.10 | 0.50 | 0.10 | 5.00 | 1.974 | 4.89 | 2.320 | 5.72 | 3.430 | 8.28 | 2.726 | 6.82 |
| 35 | 0.20 | 0.20 | 0.50 | 0.10 | 5.00 | 1.974 | 4.89 | 2.305 | 5.68 | 3.411 | 8.24 | 2.722 | 6.81 |
| 36 | 0.30 | 0.30 | 0.50 | 0.10 | 5.00 | 1.974 | 4.89 | 2.292 | 5.65 | 3.388 | 8.19 | 2.715 | 6.80 |
| 37 | 0.10 | 0.10 | 0.00 | 0.20 | 5.00 | 1.974 | 4.89 | 2.258 | 5.57 | 2.752 | 6.76 | 2.437 | 6.14 |
| 38 | 0.20 | 0.20 | 0.00 | 0.20 | 5.00 | 1.974 | 4.89 | 2.239 | 5.53 | 2.748 | 6.75 | 2.435 | 6.14 |
| 39 | 0.30 | 0.30 | 0.00 | 0.20 | 5.00 | 1.974 | 4.89 | 2.229 | 5.50 | 2.751 | 6.76 | 2.437 | 6.14 |
| 40 | 0.10 | 0.10 | 0.25 | 0.20 | 5.00 | 1.974 | 4.89 | 2.280 | 5.62 | 2.927 | 7.16 | 2.511 | 6.32 |
| 41 | 0.20 | 0.20 | 0.25 | 0.20 | 5.00 | 1.974 | 4.89 | 2.264 | 5.58 | 2.930 | 7.16 | 2.512 | 6.32 |
| 42 | 0.30 | 0.30 | 0.25 | 0.20 | 5.00 | 1.974 | 4.89 | 2.253 | 5.56 | 2.930 | 7.16 | 2.512 | 6.32 |
| 43 | 0.10 | 0.10 | 0.50 | 0.20 | 5.00 | 1.974 | 4.89 | 2.303 | 5.68 | 3.092 | 7.53 | 2.583 | 6.49 |
| 44 | 0.20 | 0.20 | 0.50 | 0.20 | 5.00 | 1.974 | 4.89 | 2.289 | 5.64 | 3.079 | 7.50 | 2.579 | 6.48 |
| 45 | 0.30 | 0.30 | 0.50 | 0.20 | 5.00 | 1.974 | 4.89 | 2.277 | 5.62 | 3.063 | 7.46 | 2.574 | 6.47 |
| 46 | 0.10 | 0.10 | 0.00 | 0.30 | 5.00 | 1.974 | 4.89 | 2.242 | 5.53 | 2.594 | 6.39 | 2.371 | 5.99 |
| 47 | 0.20 | 0.20 | 0.00 | 0.30 | 5.00 | 1.974 | 4.89 | 2.224 | 5.49 | 2.590 | 6.38 | 2.369 | 5.98 |
| 48 | 0.30 | 0.30 | 0.00 | 0.30 | 5.00 | 1.974 | 4.89 | 2.215 | 5.47 | 2.593 | 6.39 | 2.371 | 5.99 |
| 49 | 0.10 | 0.10 | 0.25 | 0.30 | 5.00 | 1.974 | 4.89 | 2.264 | 5.59 | 2.732 | 6.71 | 2.429 | 6.13 |
| 50 | 0.20 | 0.20 | 0.25 | 0.30 | 5.00 | 1.974 | 4.89 | 2.249 | 5.55 | 2.734 | 6.71 | 2.430 | 6.13 |
| 51 | 0.30 | 0.30 | 0.25 | 0.30 | 5.00 | 1.974 | 4.89 | 2.240 | 5.53 | 2.734 | 6.72 | 2.430 | 6.13 |
| 52 | 0.10 | 0.10 | 0.50 | 0.30 | 5.00 | 1.974 | 4.89 | 2.287 | 5.64 | 2.862 | 7.01 | 2.486 | 6.26 |
| 53 | 0.20 | 0.20 | 0.50 | 0.30 | 5.00 | 1.974 | 4.89 | 2.275 | 5.61 | 2.853 | 6.99 | 2.483 | 6.25 |
| 54 | 0.30 | 0.30 | 0.50 | 0.30 | 5.00 | 1.974 | 4.89 | 2.264 | 5.59 | 2.842 | 6.96 | 2.479 | 6.24 |
| 55 | 0.10 | 0.10 | 0.00 | 0.10 | 10.00 | 1.974 | 4.89 | 2.561 | 6.27 | 3.924 | 9.36 | 2.929 | 7.29 |
| 56 | 0.20 | 0.20 | 0.00 | 0.10 | 10.00 | 1.974 | 4.89 | 2.520 | 6.18 | 3.917 | 9.35 | 2.926 | 7.29 |
| 57 | 0.30 | 0.30 | 0.00 | 0.10 | 10.00 | 1.974 | 4.89 | 2.497 | 6.12 | 3.925 | 9.37 | 2.929 | 7.29 |
| 58 | 0.10 | 0.10 | 0.25 | 0.10 | 10.00 | 1.974 | 4.89 | 2.605 | 6.37 | 4.371 | 10.32 | 3.116 | 7.72 |
| 59 | 0.20 | 0.20 | 0.25 | 0.10 | 10.00 | 1.974 | 4.89 | 2.570 | 6.29 | 4.380 | 10.34 | 3.120 | 7.73 |
| 60 | 0.30 | 0.30 | 0.25 | 0.10 | 10.00 | 1.974 | 4.89 | 2.545 | 6.24 | 4.377 | 10.34 | 3.120 | 7.73 |
| 61 | 0.10 | 0.10 | 0.50 | 0.10 | 10.00 | 1.974 | 4.89 | 2.650 | 6.48 | 4.780 | 11.18 | 3.297 | 8.14 |
| 62 | 0.20 | 0.20 | 0.50 | 0.10 | 10.00 | 1.974 | 4.89 | 2.620 | 6.41 | 4.743 | 11.10 | 3.288 | 8.11 |
| 63 | 0.30 | 0.30 | 0.50 | 0.10 | 10.00 | 1.974 | 4.89 | 2.593 | 6.35 | 4.700 | 11.01 | 3.275 | 8.09 |
| 64 | 0.10 | 0.10 | 0.00 | 0.20 | 10.00 | 1.974 | 4.89 | 2.526 | 6.19 | 3.469 | 8.37 | 2.738 | 6.85 |
| 65 | 0.20 | 0.20 | 0.00 | 0.20 | 10.00 | 1.974 | 4.89 | 2.488 | 6.10 | 3.461 | 8.35 | 2.735 | 6.84 |
| 66 | 0.30 | 0.30 | 0.00 | 0.20 | 10.00 | 1.974 | 4.89 | 2.468 | 6.06 | 3.468 | 8.37 | 2.738 | 6.85 |
| 67 | 0.10 | 0.10 | 0.25 | 0.20 | 10.00 | 1.974 | 4.89 | 2.571 | 6.29 | 3.811 | 9.12 | 2.882 | 7.19 |
| 68 | 0.20 | 0.20 | 0.25 | 0.20 | 10.00 | 1.974 | 4.89 | 2.538 | 6.22 | 3.817 | 9.13 | 2.884 | 7.19 |
| 69 | 0.30 | 0.30 | 0.25 | 0.20 | 10.00 | 1.974 | 4.89 | 2.516 | 6.17 | 3.816 | 9.13 | 2.885 | 7.19 |
| 70 | 0.10 | 0.10 | 0.50 | 0.20 | 10.00 | 1.974 | 4.89 | 2.615 | 6.40 | 4.129 | 9.81 | 3.021 | 7.51 |
| 71 | 0.20 | 0.20 | 0.50 | 0.20 | 10.00 | 1.974 | 4.89 | 2.588 | 6.33 | 4.104 | 9.75 | 3.014 | 7.49 |
| 72 | 0.30 | 0.30 | 0.50 | 0.20 | 10.00 | 1.974 | 4.89 | 2.565 | 6.28 | 4.073 | 9.69 | 3.004 | 7.47 |
| 73 | 0.10 | 0.10 | 0.00 | 0.30 | 10.00 | 1.974 | 4.89 | 2.495 | 6.12 | 3.160 | 7.68 | 2.609 | 6.55 |
| 74 | 0.20 | 0.20 | 0.00 | 0.30 | 10.00 | 1.974 | 4.89 | 2.459 | 6.04 | 3.152 | 7.66 | 2.606 | 6.54 |
| 75 | 0.30 | 0.30 | 0.00 | 0.30 | 10.00 | 1.974 | 4.89 | 2.442 | 6.00 | 3.159 | 7.68 | 2.609 | 6.55 |
| 76 | 0.10 | 0.10 | 0.25 | 0.30 | 10.00 | 1.974 | 4.89 | 2.539 | 6.22 | 3.430 | 8.28 | 2.723 | 6.82 |
| 77 | 0.20 | 0.20 | 0.25 | 0.30 | 10.00 | 1.974 | 4.89 | 2.509 | 6.15 | 3.433 | 8.29 | 2.724 | 6.82 |
| 78 | 0.30 | 0.30 | 0.25 | 0.30 | 10.00 | 1.974 | 4.89 | 2.490 | 6.11 | 3.434 | 8.29 | 2.725 | 6.82 |
| 79 | 0.10 | 0.10 | 0.50 | 0.30 | 10.00 | 1.974 | 4.89 | 2.584 | 6.32 | 3.684 | 8.84 | 2.833 | 7.07 |
| 80 | 0.20 | 0.20 | 0.50 | 0.30 | 10.00 | 1.974 | 4.89 | 2.559 | 6.27 | 3.667 | 8.80 | 2.827 | 7.06 |
| 81 | 0.30 | 0.30 | 0.50 | 0.30 | 10.00 | 1.974 | 4.89 | 2.538 | 6.22 | 3.645 | 8.76 | 2.820 | 7.04 |
| *W_in_*: wash-in period, a period from the start of a given pandemic wave to the time when the anxiety disorder incidence peaks during the wave.  *W_out_*: wash-out period, a time between the moment the elevated anxiety disorder incidence starts to decline and the moment when the incidence is no longer raised higher than what would be expected from the historical trends.  *Δ*: delay, during which the COVID-19 incidence starts to subside while the anxiety disorder incidence remains elevated.  *h_0_*: the magnitude of an anxiety disorder incidence increase.  *λ*: decay constant at which *h_0_* gradually diminishes from one wave to another. | | | | | | | | | | | | | |
